# AI voices reduce cognitive activity? A psychophysiological study of the media effect of AI and human newscasts in Chinese journalism

**DOI:** 10.3389/fpsyg.2023.1243078

**Published:** 2023-11-23

**Authors:** Chen Gong

**Affiliations:** School of Journalism, Fudan University, Shanghai, China

**Keywords:** AI journalism, AI newscast, brain response, media psychophysiology, human-computer interaction

## Abstract

Artificial Intelligence (AI) has been widely utilized in automated journalism writing and broadcasting in recent years. However, few systematic studies have been conducted on the differences in brain activation between human and artificial voices in newscasts. This paper aims to investigate the psychophysiological effects of the media in Chinese contexts when different agents (AI or human) broadcast different types (emotional/neutral) of news. Comparing the electrophysiological data of the participants’ EEG while listening to different newscast agents revealed that brain activity responses were greater when listening to a human newscast than to an AI voice newscast. And β bands in left posterior temporal lobe were significantly different, suggesting that participants’ brain were better at processing, comprehending auditory information, and storing working memory when listening to a human reporter than when listening to a voice generated by AI. Moreover, the ERP results and the interaction effect of content valence and agent voice demonstrated that the human voice generated greater cognitive effect, which may reflect participants’ trust in the credibility and fluency of the human voice news. This study demonstrates the importance of further research into cognitive effects of AI journalism.

## Introduction

Intelligent voice applications have become increasingly popular due to the rapid development of artificial intelligence technology. The extensive integration of synthesized speech with news products has produced a vast quantity of audio and video content that has a increasing impact on individuals, such as intelligent broadcasts in news clients and virtual anchors in media organizations. In recent years, the study of the mediating effects of the reception of synthesized speech on the dissemination of information has emerged in fields such as communication, human-computer interaction, and others.

Carlson (2015) argued that there is an epistemological need to investigate whether the rise of AI in the news industry will result in a decline of human judgment. Some researchers conducted a series of experiments and discovered that participants’ sense of belief increased when they read allegedly AI-written news articles (Henestrosa et al., 2022). In addition, numerous researchers have investigated AI broadcast news from the perspectives of social presence and social trust (Lee et al., 2020; Heiselberg, 2021; Hofeditz et al., 2021). Researchers have discovered that the greater the audience’s use of social media, the higher they rate AI-generated news (Hofeditz et al., 2021). Listeners’ feedback is positive when users hear an AI’s voice that resembles their own social image, even if the voice is obviously artificial (Heiselberg, 2021). And social trust and frequency of discussion are positively correlated with AI news credibility (Lee et al., 2020). Although some argue that the comparison between computer-assisted broadcast journalism and computational journalism is a feature over time as opposed to a comparison of contemporaneous practice (Coddington, 2015), AI journalism and computational journalism should be discussed separately. Nonetheless, numerous studies have compared AI news articles or audio to traditional journalistic news.

In 2016, Oxford Dictionaries named “post-truth” the Word of the Year. The expression of emotion and personal beliefs influences public opinion more than the presentation of objective facts (McIntyre, 2018). The audience’s perception of objective facts is heavily influenced by emotional expressions in a “post-truth” context. And prior to the advent of artificial intelligence in mass communication, it was proposed in 2004 to use psychophysiological measures to examine the effects of news media (Kallinen and Ravaja, 2007). To examine the effect of emotional expressions in news contents on individuals’ cognitive activities, researchers selected texts of the same news event broadcasted in two different ways (emotional/neutral expressions) as experimental material and measured the effect of this cognitive communication using specialized equipment. For instance, the *Neurojour* project (Heiselberg, 2021) is an innovative pilot project within the field of journalism research that tests psychophysiological measures. In a separate study, participants in both groups (neural voices: text-to-speech technology supported by neural networks; and human reporter voices) evaluated “neural news” with particular attention to voice emotion (Heiselberg et al., 2022). And in Heiselberg (2021)’s article, they expressed concern that as AI broadcasts get closer to humans, “The Uncanny Valley” (see Mori et al., 2012) could occur, which is consistent with Clark et al.’s (2021) earlier research. Similarly, comparing emotionally charged and non-charged news broadcasts, a study found that emotion contributes to individuals’ susceptibility to false information (Martel et al., 2020). This finding suggests that interventions designed to reduce the emotional intensity of the public’s news media consumption could be expected to reduce fake news beliefs.

Therefore, previous research suggests that content valence may have a significant effect on cognitive response. In other words, regardless of agent type, emotionally charged news will elicit stronger cognitive responses from humans than neutral news. And the emotional content broadcasted by AI voices may produce the Uncanny Valley phenomenon, and in this case, an agent voice by content valence interaction effect will be observed. Thus, we ask if agent voice and content valence will have an interactive effect (RQ1). And to date, numerous experimental studies have investigated the effects of AI newscasts on listeners, but few have examined how listeners perceive the response (Kim et al., 2022). How do cognitive responses differ between AI-generated and human-voiced audio news reports (RQ2)? Overall, we find that there is a dearth of similar research in the Chinese journalism, mostly in terms of qualitative reflections, but no mature research on the psycho-cognitive aspects of human interaction with different agents through experimental or other quantitative means. This study will examine the brain activity of listeners to determine the effect of AI news audio versus human news audio, as well as the interaction between the media agent effect and news valence (emotional versus neutral news). And for the first time, psychophysiological experiments will be used to research in this topic, which may establish a paradigm for related study in Chinese contexts in the future.

## Literature review

In recent years, the development of artificial intelligence (AI) and its application across a variety of academic institutions and industries has been one of the fastest-growing areas of technology. Besides, the news media industry is an early adopter of AI, adapting it to journalism’s data, algorithms and automation (Broussard, 2018; Weber and Kosterich, 2018; Diakopoulos, 2019), despite the fact that the use of computers in journalism dates back to the 1950s and that Philip Meyer is considered as the godfather of computer-assisted reporting (Coddington, 2015). AI journalism has emerged in recent years, modeled after the social sciences through empirical methods, primarily investigation and content analysis, as well as efficient statistical analysis, in order to achieve more definitive answers to news writing or automated audio playback. In addition, researchers consistently approach technology as a communicator, view journalism as a human-machine social process, and discuss the broader ontological issues of automated news technologies (Lewis et al., 2019).

### AI-automated journalism research

As newer media and communications technologies incorporate higher levels of machine learning, AI has become an integral component of the journalism concept. Scholars of the media must consider the fact that an increasing number of intelligent entities are mediating content and that the news media is no longer merely a dull conduit between the journalist and the recipient (Sundar, 2020). Zheng et al. (2018) investigated the cross-cultural perceptions of automated news reporting among Chinese and American users. Unlike human journalist reporting, AI reporting focused on three types of news stories: economic news, sports news, and breaking news. In the United States, users reported that AI-generated news were of lower quality than human-written news, while it was opposite in China. And according to a number of studies, this phenomenon results from the interaction between authorship and the current form of journalistic objectivity (Zheng et al., 2018). This is similar to the conclusion of Henestrosa et al. (2022), whose three experimental studies found no difference between AI-written and human-written texts in terms of perceived credibility and trustworthiness. When news was objectively written or broadcast by AI, however, the credibility of its source and message increased. In other words, Zheng et al.’s (2018) experiments revealed that neutral news written or broadcast by AI elicited the same response from audiences as news written or broadcast by human journalists. There are also academics who employ qualitative research techniques (Heiselberg et al., 2022). On the basis of in-depth qualitative interviews, their reception analysis (*n* = 12) investigated how Danish radio listeners perceive the credibility and news content of neural readers when they hear complete news broadcasts through neural voices. It has also been demonstrated experimentally that there are no differences in perceptions of credibility, communication skills, or interaction intentions between AI and human Twitter agents (Edwards et al., 2014), and the same conclusion can be drawn from Clerwall (2014), whose findings suggest that while AI-generated content and audio is considered boring and descriptive, it is also perceived as objective. And some academics have expanded their research on AI news writing or broadcasting by designing structural equation models and conducting experiments to establish a theoretical basis for the impact of AI social robot emotions on the relationship between normative beliefs and functional traits (Shin, 2021).

### Credibility of AI news

Some researchers have combined the concept of AI newscasts and misleading or fake news. Pennycook and Rand (2021) conducted a literature review on the reasons why people believe and spread highly misleading news online. The authors conclude that these behaviors are associated with heuristics. AI could successfully encourage social media users to prioritize accuracy. For instance, crowdsourced accuracy ratings could be used to improve social media ranking algorithms. Social trust is positively correlated with the credibility of AI news broadcasts, as is the frequency of discussion, according to Lee et al. (2020). In addition, Lee et al. (2020) stated that scholars present a more nuanced picture of how human and AI news writers influence the evaluation of news content. Using the New York Times (NYT) as an example, they discovered that the use of AI-based NYT articles decreased perceptions of the source’s credibility and expertise. Moreover, according to studies, even if AI broadcasts are accurate, their reception alters the emotional state of listeners. AI newscasts typically receive higher ratings than those produced by human journalists (Graefe et al., 2018). And in terms of ratings, a meta-analysis revealed that participants only gave higher ratings and higher quality when they knew they were receiving news from humans as opposed to AI (Graefe and Bohlken, 2020). Consumers find human journalist newscasts more enjoyable than computer-generated content, according to a study (Graefe et al., 2018). Graefe et al.’s (2018) experiment involved various measures of news broadcast quality, was conducted in different countries, and was based on a large sample of participants. Their findings corroborate those of two earlier studies (Clerwall, 2014; Van der Kaa and Krahmer, 2014).

### AI voice broadcasting

Academics examine more than AI news writing when AI is used in news broadcasting, as little is known about how listeners will react to AI-delivered audio (Kim et al., 2022). In the context of AI broadcasting weather news, Kim et al.’s (2022) study compared perceptions of AI news coverage to perceptions of human news agencies. There was no difference between types of newsreaders in terms of the information listeners sought regarding their intentions and behavior intentions. Voice is a characteristic that distinguishes AI from human newsreaders (e.g., AI voice vs. human voice). Although machine voices can closely imitate human voices, they are not identical (Kim et al., 2022). Over the past century, it has been demonstrated that the human voice has distinctive acoustic properties (Nass and Steuer, 1993), or that humans are more sensitive to human voices than machine sounds. And Xu (2019) found that subjects with prior experience interacting with AI responded more strongly to human audio than to AI audio. Overall, although the different effects remain controversial, the existing research narrative is clear on how people perceive AI-synthesized sounds and how they react to AI news players, which partially lays the theoretical groundwork for our research.

### Media psychophysiological research on AI newscast

As researchers examined the psychological impact of AI news broadcasts on listeners, they began conducting psychophysiological rather than traditional communication experiments. Even though AI did not exist in 2006, scholars investigated newspapers and online newspapers (online media). Using an eye-tracking model, Bucher and Schumacher (2006) analyzed the agenda-setting process of print and online newspapers for their audiences, examining how the type of media and form of news influenced attention and selectivity. The later years of the study have become progressively more diverse as a result of technological progress, which is most evident in the modernization of psychophysiological equipment. *Neurojour* is an example of a pilot study focusing on brain processing of digital news (Heiselberg, 2021). This project involves evaluating four distinct psychophysiological techniques, including EEG, eye tracking, EDA, and facial coding. The authors view this as a methodological update to journalism research, as these methods are rarely employed and traditional journalism research focuses on the conscious content of communication, such as the pragmatic dimensions of news use. Due to this limitation, the academic community has neglected to investigate audience emotions beyond the cognitive and pragmatic dimensions of news (Costera Meijer, 2020; Heiselberg, 2021). According to Kallinen and Ravaja (2007), while the number of studies employing psychophysiological measures in the field of human-computer interaction has grown, research on communication, media, and media interfaces remains scarce. The use of psychophysiological measures, in this case physiological results such as skin conductance, heart rate, and facial EMG, to investigate emotional psychological responses to news products is therefore a promising avenue. For instance, one study (Soroka et al., 2016) utilized sensors to record subjects’ heart rate, skin conductance, and respiratory amplitude facial muscle activation to investigate gender-specific responses to emotionally charged news content. In contrast, Seleznov et al. (2019) used more advanced EEG equipment to analyze the audience’s perception of emotional stimuli during news broadcasts. Similarly, the difference in N400 waves in the ERP experiment demonstrates that listeners can activate specific visual information when comprehending news broadcasts (Rommers et al., 2013), as well as experiments demonstrating that emotional news is more likely to excite listeners via differences in EEG signals (Liu et al., 2013), and using EEG to determine the effect of gender on AI-generated speech for newscasts.

In summary, the existing literature introduces various cross-disciplinary approaches to the study of the media effects of AI newscasts, but there has not been a systematic use of psychophysiological measures to conduct comparative studies of multiple factors (AI news audio versus human audio, emotional versus non-emotional news), so in conjunction with the findings of the literature review, we formulated the two hypotheses outlined in the preceding section and conducted experiments to test them.

## Hypotheses

We expect that because AI synthesized voices in current AI technology are still unable to effectively handle special cases of accented rhythm, alliteration, and Chinese Pronunciation Erhua in its contexts, human voices may have more syllable and accent processing shifts than AI-synthesized voices, thereby expressing more emotion and cueing and eliciting stronger cognitive-emotional feedback from listeners, as reflected in this study primarily in in brainwave activity. Given that there have been articles using psychophysiological methods to study AI newscast (Bucher and Schumacher, 2006; Kallinen and Ravaja, 2007; Seleznov et al., 2019; Heiselberg, 2021; Heiselberg et al., 2022), our first research hypothesis is that human-voiced news broadcasts induce greater EEG activity and cognitive activation in listeners than AI-synthesized voices, and consequently have greater cognitive communication effects (Hypothesis 1).

Current studies comparing AI voices with human voices have primarily used gender as the independent variable and conducted between-group analyses through experiments (e.g., Soroka et al., 2016; Kim et al., 2022), but the field of communication has not yet seen a comprehensive study of different emotional news types through psychophysiological experiments, we propose hypothesis 2: We expected that both emotionally-rich news content and neutral news would result in higher brain activity caused by the human voice broadcast than AI, which was mainly reflected in higher EEG amplitude than that of the experimental group (AI newscasts), potentially longer frequency domain duration, and greater activation of certain cortical areas of the brain, etc., in the experiment. In other words, regardless type of the news, human voices have a greater impact on the media than AI voices (Hypothesis 2).

## Materials and methods

### Experiment design

The study includes a pre-experiment session and a formal experiment session. The pre-experiment was a within-subject design with the attempt of replicating previous studies (Waddell, 2018; Heiselberg et al., 2022) that found news attributed to a machine newscaster was perceived as less credible than news attributed to a human newscaster, even though the news was read by AI voices.

The formal experiment session employed a 2 (agent: AI synthesized voices and human voices) × 2 (content valence: emotional and neutral) mixed factorial design. The agent factor was a between-group variable and content valence was a within-group variable. The formal experiment contained two phases. Phase 1 asked participants to listen to one neutral news clip and Phase 2 asked participants to listen to one neutral and one emotional clip.

### Ethical approval

The experiment conformed to The Code of Ethics of the World Medical Association (Declaration of Helsinki). This study was also supported by the Third People’s Hospital of Mianyang City and approved by the Human Investigations Committee of it.

### Informed consent

All participants signed an informed consent form and were compensated appropriately after the experiment.

### Stimulus materials

For the pre-experiment, 10 neutral television news’ audio clips were selected and their corresponding AI news was generated using an AI newscaster generation website.^1^ For the formal experiment, in Phase 1, 10 news audio clips (different from the pre-experiment) were selected and their corresponding AI news was generated using the aforementioned online generator. In Phase 2, a total of 20 news audios (different from the pre-experiment and Phase 1) were selected, with 10 emotional and 10 neutral audios. Accordingly, 20 AI news clips were generated using the same procedure described above. All human and AI newscasters were female. Each audio clip was 30-s long. There was no loss of sound sample bands or compression when intercepting the sound clips.

### Participants

This study recruited a total of 30 participants, mostly young adults between the ages of 18 and 40, with 16 female and 14 male participants. The number of participants is also roughly similar to the number of subjects in the current state-of-the-art EEG dataset. And since this paper is an effort to actively introduce a psychophysiology experiment or paradigm into the field of cognitive communication research, and a series of subsequent work will be conducted accordingly, so this number of participants is reasonable at this stage.

All subjects were right-handed, had normal hearing and vision, had not sustained a head injury in the week preceding the experiment, and had not taken psychostimulants or other drugs affecting central nervous function. In addition, neither they nor their relatives had a history of psychiatric or neurological disorders or a related family history (e.g., epilepsy). All participants signed an informed consent form and were compensated appropriately after the experiment.

Before the experiment, participants were required to complete the Beck Anxiety Inventory (Beck et al., 1993) and the Depression Inventory (Beck and Beamesderfer, 1974) in the laboratory. The authors then screened participants’ mood states based on their scale scores, and abnormal individual data were excluded. Nonetheless, the results demonstrated that none of the participants scored at or above the mild depression level on the depression scale, and none of the participants displayed clinical symptoms of anxiety or depression during the formal experiment, indicating that the experiment data was convincing.

### Experimental procedure

Upon signing the informed consent form, the standardized preparation was followed. In the pre-experiment, each participant listened to one news clip broadcasted by human and another different news clip broadcasted by AI. After listening to each newscast, participants were asked to rate its credibility (an individual’s judgment of the veracity of the broadcasted content), fluency (whether the news is presented in a continuous manner and the particular rhythms, such as pauses and accents, are well managed), and comprehensibility (whether the meaning conveyed by the news audio is clear and unambiguous) on a 5-point Likert scale (Likert, 1932), the scale evaluation metrics in this pre-experiment were derived from Appelman and Sundar’s (2016) and Waddell’s (2018) experiments.

**FIGURE 1:**
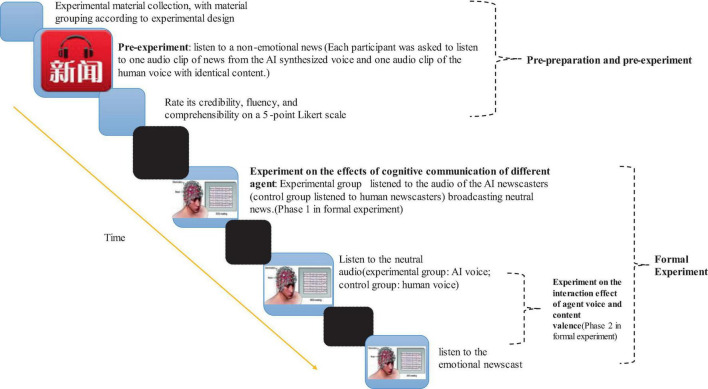
Experimental flow diagram. For the pre experiment, 10 neutral television news’ audio clips were selected and their corresponding AI news was generated using an AI newscaster generation website. or the formal experiment, in hase 1, 10 news audio clips (different from the pre experiment) were selected and their corresponding AI news was generated using the aforementioned online generator. In hase 2, a total of 20 news audios (different from both the pre experiment and hase 1) were selected, with ten emotional and ten neutral audios.

In the formal experiment, participants were randomly assigned to the AI-voice group (experiment group) and the human-voice group (control group). Each participant listened to one neutral news clip. After a 10-min break, they were then instructed to listen to one neutral news clip, followed by one emotional news clip. See Figure 1 for the detailed process during both experiment stages.

### Data processing and analysis

The EEG data were recorded using specialist equipment, which is 33 electrodes, arranged in a 10–20 system of electrode positions, with a sample rate of 256 Hz. A Common Mode Sense (CMS) and Driven Right Leg (DRL) electrode were used to provide an active ground. The left mastoid was used as the reference electrode, which was converted to a bilateral mastoid average reference for off-line analysis.

Heavy contamination of EEG activity by eye movements, blinks, muscle, heart, and line noise poses a serious problem for EEG analysis and interpretation (Iwasaki et al., 2005). We manually removed EEG errors caused by blinking, eye movement, and head movement for the specific experimental conditions of the subjects in this experiment. Specifically, the following criteria are used to reject artifacts during EEG frequency domain analysis:

(1) Blinking components: High energy at low frequencies, randomly distributed, with components ranked high in the frequency domain analysis diagram, distributed as small squares in the front of the human brain in the frequency domain analysis diagram.

(2) Eye movement components: It is red-blue relative and distributed on both sides of the front of the human brain in the EEG frequency domain analysis map. It possesses high energy at low frequencies and is dispersed in long strips.

(3) Head movement components: In the EEG frequency domain analysis map, the brain’s spectral energy is concentrated. Individual EEG energy curves display a significant drift. We simultaneously use ICA to remove artifacts from EEG. ICA is a feature extraction technique for transforming multivariate random signals into signals with mutually independent components (Subasi and Ismail Gursoy, 2010). ICA was initially proposed by Bell and Sejnowski (1995), and their original interpretation involved a complex idea of infomax principal, a concept that is generally unnecessary in contemporary ICA, and we use maximum likelihood techniques to determine. Assume that the probability distribution of source *s_j_* is *p_s_*(*s*_*j*_) and the sources are independent of each other, thus the joint probability distribution of the overall EEG frequency domain analysis signal is


(1) $$p\,\left(s\right)=\prod_{j=1}^{d}p_{s}\,\left(s_{j}\right)$$

Considering that *x* = *As* = *W*^−1^*s*↔*s* = *Wx*, equation (1) can be transformed into


(2) $$p\,\left(s\right)=\prod_{j=1}^{d}p_{s}\,\left(W\,x\right)\,\left|W\right|$$

The only remaining variable is the probability density *p_s_*(*s*_*j*_). Probability density is the derivative of the cumulative distribution function (CDF), and if the exact probability distribution of the source signal is unknown, a common choice for the CDF is the Sigmoid $g\,\left(x\right)=\frac{1}{1+e^{-x}}$, which rises gradually from 0 to 1 (Tuerlinckx, 2004). According to the probability density function formula:


(3) $$p\,\left(x\right)=g^{{}^{\prime }}\,\left(x\right)=g\,\left(x\right)\,\left(1-g\,\left(x\right)\right)$$

Given the training set {*x*^(*i*)^ : *i* = 1, 2, ⋯, *n*}, we can derive likelihood(L) and take the logarithm of the result:


(4) $$L=\prod_{n=1}^{n}\prod_{j=1}^{d}p_{s}\,\left(W\,x\right)\,\left|W\right|\,\underset{}{\Rightarrow }\,l=\text{log}\,L=\sum_{n=1}^{n}\sum_{j=1}^{d}\text{log}\,p_{s}\,\left(W\,x\right)\,\left|W\right|$$

$$=\sum_{n=1}^{n}\sum_{j=1}^{d}\text{log}\,p_{s}\,\left(W\,x\right)+\text{log}\,\left|W\right|$$

$$=\sum_{n\,1}^{n}\left(\sum_{j\,1}^{d}\text{log}\,g^{{}^{\prime }}\,\left(W_{j}^{T}\,x^{\left(i\right)}\right)+\text{log}\,\left|W\right|\right)$$

To maximize *W*, the iterative formula for stochastic gradient ascent (1 sample) can be derived, where the matrix derivative of the determinant is used, and the final derivation is


(5) $$W:=W+\alpha \left(\left[\begin{array}{c} 1-2\,g\,\left(w_{1}^{T}\,x^{\left(i\right)}\right)\\ 1-2\,g\,\left(w_{2}^{T}\,x^{\left(i\right)}\right)\\ \cdots \\ 1-2\,g\,\left(w_{d}^{T}\,x^{\left(i\right)}\right) \end{array}\right]x^{\left(i\right)\,T}+{\left(W^{T}\right)}^{-1}\right)$$

After the algorithm converges, we then computed *s* to recover the original sources and extracted artifacts from EEG data using ICA. Finally, we extracted the power spectral density (PSD) values of delta (1–4 Hz), theta (5–7 Hz), alpha (8–13 Hz) and beta (14–30 Hz) bands on the electrode channel using Fast Fourier Transform (FFT) and conducted a series of subsequent analyses.

## Result

### Pre-experimental results

*T*-test revealed significant differences between AI and human voices in terms of content credibility and broadcasting fluency. Human voices were perceived being more credible and fluent than AI voices (see Table 1).

**TABLE 1:** Paired *t*-test results for pre-experiment.

				95% CI for Cohen’s d		
AI versus human voice	*t*	df	*p*	Cohen’s d	Lower	Upper
Credibility	−4.000	9	0.003*	−1.265	−2.092	−0.401
Fluency	−3.873	9	0.004*	−1.225	−2.040	−0.373
Comprehensibility	−1.765	9	0.111	−0.558	−1.215	0.125

Student’s t-test. All variables measured on five-point, Likert-type scale where 1 = “strongly disagree,” 5 = “strongly agree.” *P < 0.05.

The pre-experimental questionnaire focused on the subjective perceptions of two types of news audio from the participants. The results revealed statistically significant differences between AI news broadcast and human news broadcast in terms of credibility: *t*(9) = −4.000, *p* = 0.003 < 0.05, 95% CI for Cohen’s *d* = [−2.092, −0.401] and in terms of fluency: *t*(9) = −3.873, *p* = 0.004 < 0.05, 95% CI for Cohen’s *d* = [−2.040, −0.373], while the difference was not significant in terms of comprehensibility: *t*(9) = −1.765, *p* = 0.111 > 0.05, 95% CI for Cohen’s *d* = [−1.215, 0.125]. Pre-experiment results provide additional evidence that the experiment is feasible. Participants perceived differences in fluency and credibility between the two types of newscasts, possibly as a result of differences in the AI’s pronunciation of certain syllables and intonation compared to that of humans. The inherently more comprehensible lexical rhythm of the news scripts may explain why the AI news did not present significant comprehensibility issues for the subjects. Moreover, syllable and intonation variations in the AI broadcast did not contribute to overall comprehensibility issues.

### Phase 1 EEG results

In Phase 1 of the formal experiment, participants were instructed to pay close attention to each speech, and each listened to single audio segments. We initially performed a spectral analysis of the experimental EEG data by averaging all the collected EEG electrode data and the resulting EEG response spectrum is displayed below. The various colors in the graph represent different levels of spectral energy, with the spectral energy increasing from low to high and the colors becoming lighter.

**FIGURE 2:**
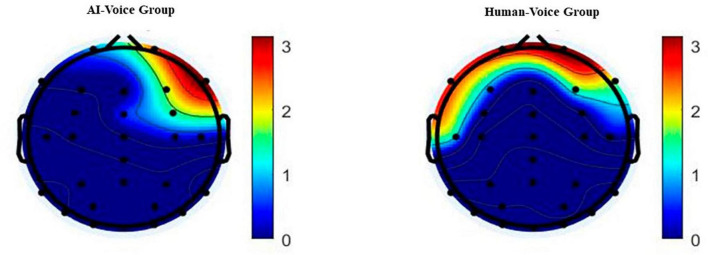
Topographical map of the EEG spectrum. Group 1 is the AI voice group, and the image is a plot of the power spectral density obtained by averaging the electrode data of the AI voice group members after the subjects listened to the AI newscast. ight shows the uman voice group, where the experimental material is the audio of human newscasters broadcasting the news, and the data processing operation is the same as the AI-voice group.

**FIGURE 3:**
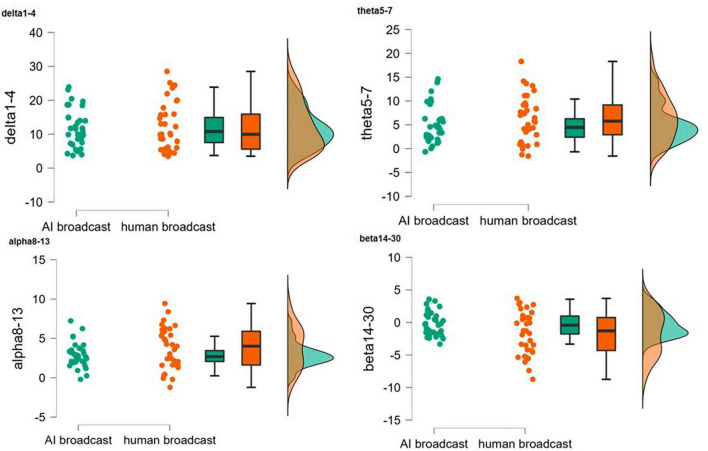
Cloud and rain map of the four types of EEG waves.

The primary conclusion from Figure 2 is that the prefrontal lobe of the brain differs between the experimental group and the control groups. The mean voltage over F3 electrode was submitted to a *t*-test between AI voice and human voice group. *T*-test analysis confirmed the agent-related differences (mean amplitude ± SD) in AI voice: −1.377 ± 3.06 μV and human voice: −0.293 ± 1.856 μV (*t* = −3.463, *p* = 0.002 < 0.005, 95% CI Cohen’s *d* = [−0.97, −0.227]). For specifics on other electrodes, please refer to the details in https://osf.io/wcemb/. The results are consistent with Manfredi et al. (2021)’s and Song et al. (2022)’s research, which suggests the asymmetry in frontal EEG activity is associated with experiential pleasure. Then, we analyzed four common waves (α, β, θ, δ) in human brain electrical activity in the prefrontal from both groups by using independent samples *t*-test, and the following results emerged:

The cloud and rain plots (see Figure 3) and *t*-tests (Table 2) revealed significant differences (*t*(64) = 2.04, *p* = 0.046 < 0.05, 95% CI for Cohen’s *d* = [0.01, 0.991]) in the EEG response spectrum at beta-band frequencies (14 Hz–30 Hz) between AI and human newscasters. This is consistent with (Engel and Fries, 2010) research, who proposed that the activity in the β band is associated with an active mental state and is increased in the context of attentional focus and emotional arousal. Combined with Spielberg et al. (2008)’s study, we can roughly observe the differences in emotional processing between two groups of subjects while listening to the audio, and these will be deeply examined Phase 2. And for the result of δ waves (*t*(64) = −0.243, *p* = 0.809 > 0.05, 95% CI for Cohen’s *d* = [−0.542, 0.423]), θ waves (*t*(64) = −1.087, *p* = 0.281 > 0.05, 95% CI for Cohen’s *d* = [−0.751, 0.218]), α waves (*t*(64) = −1.667, *p* = 0.1 > 0.05, 95% CI for Cohen’s *d* = [−0.897, 0.079]), neither is statistically significant. The experimental results also confirm a difference in the intensity of the subjects’ low-frequency neural activity in the brain, primarily β waves, when listening to and comprehending AI-generated as opposed to human sounds. We then extracted the β band from the two EEG data sets and mapped the results to the EEG spectral topography using independent samples *t*-test, as follows:

**TABLE 2:** Independent samples *t*-test for four waves.

						95% CI for mean difference			95% CI for Cohen’s d	
	*t*	df	*p*	Mean difference	SE difference	Lower	Upper	Cohen’s d	Lower	Upper
Delta 1–4	0.243	64	0.809	−0.386	1.588	−3.559	2.787	0.060	0.542	0.423
Theta 5–7	1.087	64	0.281	−1.184	1.089	−3.361	0.992	0.268	0.751	0.218
Alpha 8–13	1.667	64	0.100^a^	−0.900	0.540	−1.979	0.178	0.410	0.897	0.079
Beta 14–30	2.040	64	0.046^a^	1.357	0.665	0.028	2.686	0.502	0.010	0.991

Student’s *t*-test. ^a^Levene’s test is significant (*p* < 0.05), suggesting a violation of the equal variance assumption.

As the temporal lobe is primarily responsible for higher neural activities such as hearing, language comprehension, smell, memory, and imagination (Squire and Zola-Morgan, 1991), the difference in β waves between the two groups observed in the right posterior temporal lobe is consistent with Squire and Zola-Morgan (1991). As the number of electrodes was limited, there were insufficient electrodes to collect EEG data in the parietal, temporal, and occipital junction regions. Consequently, there may have been some bias in the region of the brain that displayed significant differences in the images. However, it is still possible to conclude that human newscasters were more engaging than AI newscasts. This may be reflected in the fact that partially rusty paralinguistic cues such as intonation, pauses, and rhythm in the synthetic voice reduce cognitive processing of speech content in the human brain, thereby diminishing the subjects’ capacity to allocate attention and cognitive resources.

### Phase 2 ERP results

According to the experimental design, the participants took a ten-minute break after listening to the first news audio before beginning the second stage. The second step involved listening to two distinct newscasts read by a human or AI. The primary objective of this experiment was to examine the neural responses of the subjects to neutral and emotional news broadcast by diverse news representation groups (humans or AI). As for the participants, they were instructed to listen to neutral news first, then emotional news, to avoid emotional fluctuations that could lead to measurement errors if emotional news was heard first.

**FIGURE 4:**
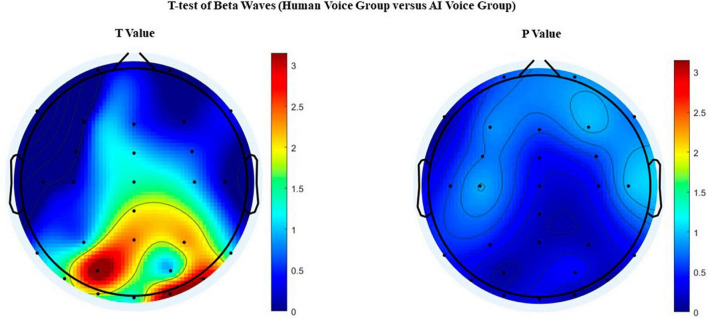
EEG topographic mapping after *t*-test of beta waves. The beta waves of the two groups were significantly different in the left parietal, right posterior temporal lobe and other regions.

**FIGURE 5:**
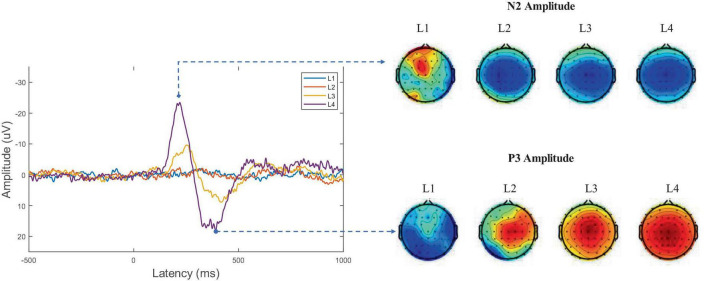
Comparative analysis of ERP for four experimental conditions. L1 and L3 are the images obtained by the AI voice group, while L2 and L4 are obtained by the human voice group. pecifically, L1 ubjects listen to non emotional audio broadcast by AI newscasters; L2 ubjects listen to non emotional audio broadcast by human newscasters; L3 ubjects listen to emotional audio broadcast by AI newscasters; L4 ubjects listen to emotional audio broadcast by human newscasters. The spectrograms on the right correspond to the 200 and 300 moments, and from left to right are images of L1, L2, L3, L4.

Throughout the experiment, we recorded the brain waves of the subjects. After processing the raw data, a comparative analysis of the four types [(human or AI newscaster) * (emotional or neutral news)] of listening to news audio was conducted using event-related potentials (ERP) time domain analysis, Figure 4 illustrates the EEG time domain analysis obtained for one of the experimental conditions, where the horizontal axis represents the moment of audio playback and the vertical axis represents the brain’s physiological voltage; note that negative voltage is positive with respect to the vertical axis in this case. Consequently, Figure 5 illustrates the waveform that appears around 200 ms is denoted N200 and the waveform that appears around 370 ms is denoted P300. The N200 is a subcomponent of the N2 component, which is responsible for categorizing stimuli and the processes preceding working memory storage. While the N2 is typically divided into at least three subcomponents, including one pre-central (pre) component associated with the detection of novelty or mismatch from perceptual templates when attending to evoked stimuli, a second pre-central component associated with cognitive control (including response inhibition, response conflict, and error monitoring), and one or two posterior N2 associated with certain aspects of visual attention. P3 is generally caused by inconsistent sounds, indicating a double dissociation between the physical and semantic properties of the sound (Manfredi et al., 2021). And P3 is associated with attention-related memory processing, as the amplitude of P3 increases as participants exert more effort on a given task. Moreover, this is consistent with numerous experimental brain electrophysiology specifications (Nobre et al., 1994; Kopp et al., 1996; Patel and Azzam, 2005).

The results of the experiment revealed that the subjects exhibited the strongest EEG activity when the control group heard a human newscaster broadcast emotionally charged news text, and a similar response when the experimental group heard an AI newscaster broadcast emotionally charged news text, but with weaker EEG activity. For neutral news, there was little difference between the subjects’ responses to hearing a human or an AI newscaster, as the bands of the two types of neural activity were difficult to distinguish and overlapped.

**FIGURE 6:**
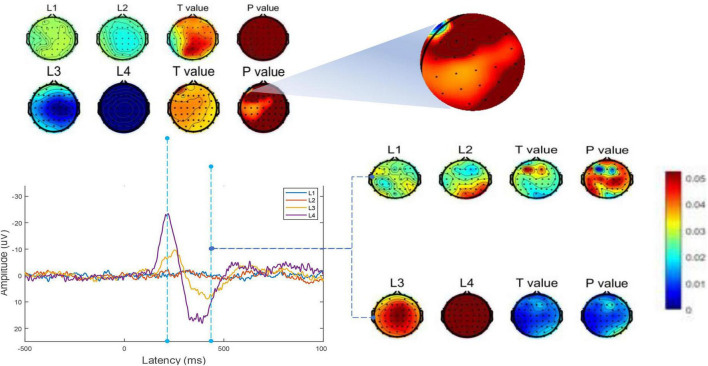
Independent samples *t*-test for brain activity in ERP. For different broadcast voice agents for the same emotional news (emotional/non-emotional) at n200 and p300 moments.

We then recorded moments N200 and P300 and conducted independent samples *t*-tests for the two participants groups (moment N200: L3 and L4 conditions, implying a comparison between AI synthesized voices and human voices broadcasting emotional news; moment P300, L1 and L2 condition, L3 and L4 condition, meaning a comparison between experimental and control groups). The results are shown in Figure 6.

Significant differences were found between the two experimental conditions (AI broadcasting emotional news versus human voice broadcasting emotional news) at L3 and L4, primarily in the left frontal cortex at the N200 moment, which coincided with the differences in the β wave active regions in phase 1. And as time progressed to the P300 moment, the EEG differences between the two experimental conditions described above became more pronounced, whereas there was no statistically significant difference between condition L1 and L2. The phenomenon that emotional expressions are more likely to spread in people’s daily lives may be partially explained by the fact that emotional expressions are more likely to stimulate cortical activity and entice audiences to listen to them. Emotional newscasts are inherently subjective compared to neutral objective statements, they are consistent with or in conflict with the listener’s preconceived values, beliefs or attitudes, and participants may devote more cognitive resources to processing emotional-related tasks. In conclusion, it is accurate to assert that in Chinese contexts, the mediating effect brought by AI voices broadcasting news is weaker than that of human voices, especially when broadcasting emotionally charged news, which is primarily reflected in the different effects on the cognitive activity of the listener’s brain. And a study on the correlation between Mandarin and cortical auditory evoked potentials can be found in a recent article (Chen et al., 2022).

To test the hypotheses proposing main and interaction effects, a 2(Agent: AI or human) * 2(Type of news: emotional or neutral) MANOVA was conducted by entering N200 and P300 as dependent variables. See Table 3 the following table.

**TABLE 3:** Multivariate Tests for interaction of agent and news valence.

Effect		Value	*F*	Hypothesis df	Sig.	Partial η^2^
Intercept	Pillai’s Trace	0.447	949.897^b^	2.000	<0.001	0.447
	Wilks’ Lambda	0.553	949.897^b^	2.000	<0.001	0.447
Agent	Pillai’s Trace	0.175	249.460^b^	2.000	<0.001	0.175
	Wilks’ Lambda	0.825	249.460^b^	2.000	<0.001	0.175
Type of news	Pillai’s Trace	0.427	876.297^b^	2.000	<0.001	0.427
	Wilks’ Lambda	0.573	876.297^b^	2.000	<0.001	0.427
Agent * type of news	Pillai’s Trace	0.131	177.796^b^	2.000	<0.001	0.131
	Wilks’ Lambda	0.869	177.796^b^	2.000	<0.001	0.131

Design: Intercept + Agent + typeofnews + Agent * typeofnews; ^b^Exact statistic.

The results show that the main effect of agent on ERP amplitude for both moments is significant (*F* = 249.46, *p* < 0.001, partial η^2^ = 0.175), the main effect of news type on ERP amplitude for both moments is significant (*F* = 876.297, *p* < 0.001, partial η^2^ = 0.427), and the interaction effect of agent and news type is also significant (*F* = 177.796, *p* < 0.001, partial η^2^ = 0.131).

## Discussion

In the phase 1, the EEG data of the control group (human news audio) indicated greater activation, primarily in the left prefrontal cortex (LPFC). This difference in mediated effects may be attributable to the fact that the human voice is better suited to engage the listener’s working memory while hearing the news. Working memory facilitates quick learning and memory consolidation via the hippocampus (Euston et al., 2012), as evidenced by the greater engagement of the prefrontal cortex (PFC) in memory and thought in the control group in comparison to the experimental group. In addition, as proposed by Farnsworth (2019), frontal lobe asymmetry can be used to evaluate respondents’ interest in newscasts. Frontal asymmetry reflects the tendency of one’s working memory to avoid approach news content or not (Tremblay et al., 2014; Chen et al., 2022), or to choose to interact with it or not (Sheehan et al., 2005). Physiological signals in our research (e.g., in F3 electrode, *t* = −3.463, *p* = 0.002 < 0.005, 95% CI Cohen’s *d* = [−0.97, −0.227]) also indirectly reflect this transient engagement.

For RQ1, we conducted a MANOVA on the ERP results of the four conditions (2 agent * 2 type-of-news) in Experimental Phase 2 to confirm that there was indeed an interaction between the newscast agent and the valence. Additionally, we discovered differences in the left prefrontal lobe of people participating in the Phase 1 experiment who were listening to news broadcasts from various agents by recording their EEG responses. It was discovered through additional statistical analysis that the β wave was what made the difference (*t* = 2.04, *p* = 0.046, 95% CI for Cohen’s *d* = [0.01, 0.991]), supporting hypothesis 1. As for RQ2, we aimed to improve the study for the Phase 2 experiments by determining if the discovery of news emotional types would result in new findings. ERP analysis was used to record the relatively large N2 and P3 amplitudes of the participants’ brain cognition responses during news listening. Although the auditory-induced P3 is not necessarily a biomarker of learning (Tremblay et al., 2014), however, when combined with N200, it represents the process of assigning resources to one or more memory processing tasks within a process of constrained cognitive capacity, as studies have demonstrated that verbal stimulation influences the N2 component (Oppitz et al., 2015; Tomé et al., 2015). And the greatest magnitude can be deduced from the difference waves in the two experimental settings in the area of the scalp above the auditory cortex. In addition, each of our experimental settings occurred in Chinese contexts. For example, the selected news sources are all written in Chinese. We discovered that in the Chinese context, the human voice broadcast had a stronger mediating effect than the AI-generated voice. This finding may be related to the fact that the Chinese language has more syllable, intonation, and stress variation than English, which leads to more cognitive scheduling and memory processing in the listener’s brain and supports the Song et al.’s (2022) experiment, which suggested that the human voice and the synthetic voice processed different phonemes in the English context, resulting in different N2 and P3. In summary, hypothesis 2 was supported.

In contrast, the AI and human groups had the greatest mediated effect on subjects during phase 2 when emotional news was broadcast, as measured primarily by the amplitude of fluctuations in the wave spectrum of ERP analysis. We believe a strong correlation exists between this and the accent technique. Stress is the most significant aspect of audible speech that conveys attitudes and emotions and reflects the intent of the utterance. When both the subject and stimulus are in Chinese, which can be more variable than English, there is often no standard formula for determining the emphasis. Rather, it is determined and differentiated based on the specific context and the semantic meaning to be emphasized. Therefore, the experimental group has a lower stimulus effect than the control group for the same experimental material, given the AI news anchor relies on collected audio-visual and programmed data to simulate human broadcasts. This is why the experimental group has a lower stimulus effect than the control group for the identical experimental material. The second point is that in the Chinese context, emotional texts undergo more accent processing than non-emotional texts, such as the common Chinese tone processing “wanting to be strong before being weak, wanting to be high before being low,” so emotional news broadcasts have a greater impact on both groups. As confirmed by Rommers et al. (2013), the waveforms in the ERP analysis may reflect the fact that when listening to news audio, listeners may activate specific visual information when comprehending specific words. They demonstrated, through ERP, that the brain is predictive of language processing and is not limited to semantic information.

## Limitations

Regarding the limitations and prospective of this study, this experiment’s sample size is relatively small. Additionally, the gender and age of the subjects were not strictly controlled in this study. Future research could be conducted by limiting the aforementioned conditions to specific groups of individuals, such as (1) diverse audiences in cross-cultural contexts (2) political, financial, and sports newscasts, etc. We must also need to consider the moral and ethical issues raised by AI newscasts, as the rise of automated news broadcasting in the organizational, professional, and social spheres has led to the emergence of new ethical challenges. Transparency, including the disclosure of data sources and automated algorithms, arises as an emerging ethical issue. Moreover, as AI technology develops, there will inevitably come a time when AI newscasts will sound nearly identical to human newscasts. According to prior research, the excessive use of human-like with limited communication capabilities can result in the uncanny valley (Mori et al., 2012). While the majority of uncanny valley research has focused on vision, there is a growing body of work exploring perceptual mismatches via audio (Grimshaw, 2009; Meah and Moore, 2014; Moore, 2015). In the future, we may investigate the potential perceptual tensions induced by human-like voices in news audio using physiological psychometric devices similar to those utilized in this study. Besides, exploring the applications and challenges of AI in digital platforms should also be highlighted (Lacárcel, 2022; Saura et al., 2022, 2023). In conclusion, collaborative systems that intelligently exploit and combine the strengths of human and machine agents represent the future of media.

## Conclusion

In sum, we found that listeners’ cognitive activity was greater when listening to the audio of a human voice newscast than AI synthesized voice broadcast, and the different activation of β waves (*t* = 2.04, *p* < 0.05, 95% CI [0.01, 0.991]) in these conditions suggests that people’s brain have better ability to process and understand auditory information and store working memory for human voice news than for AI-synthesized voice news. Besides, experiment demonstrates the interaction effect of news content emotion and broadcast agent (*F* = 177.796, *p* < 0.001, partial η^2^ = 0.131) on the brain activity of listeners, as the human voice is more mediated in broadcasting both emotional and neutral news than the AI synthesized voice. This difference in perceived communication effectiveness is reflected in people’s dissatisfaction and skepticism regarding the credibility (*t* = −4.0, *p* < 0.05, 95% CI [−2.092, −0.401]) and fluency (*t* = −3.873, *p* < 0.05, 95% CI [−2.040, −0.373]) of AI-generated voices used for news broadcasting. This paper will provide a preliminary analysis of the psychophysiological mediated effects of various news broadcast agents, and future research will focus on the media effects of AI news audio on specific populations.

## Data availability statement

The datasets presented in this study can be found in online repositories. The names of the repository/repositories and accession number(s) can be found below: https://osf.io/wcemb/.

## Ethics statement

The studies involving humans were approved by the Human Investigations Committee of the Third People’s Hospital of Mianyang City. The studies were conducted in accordance with the local legislation and institutional requirements. The participants provided their written informed consent to participate in this study.

## Author contributions

CG: writing—original draft, reviewing and editing, experiment conducting, and data processing.

## Footnotes

1
https://play.ht/text-to-speech-voices/newscaster-voices/
